# CYP2C19 loss-of-function variants are independent risk factors for premature cerebral infarction: a hospital based retrospective study

**DOI:** 10.1186/s12872-024-04269-0

**Published:** 2024-10-29

**Authors:** Yuliang Shi, Yuxian Yang, Miaoling Feng, Heming Wu

**Affiliations:** 1 Department of Neurology, Meizhou People’s Hospital, Meizhou Academy of Medical Sciences, Meizhou, China; 2 Department of Prenatal Diagnostic Center, Meizhou People’s Hospital, Meizhou Academy of Medical Sciences, Meizhou, China

**Keywords:** Premature cerebral infarction, CYP2C19, Triglyceride-glucose index, Dyslipidemia

## Abstract

**Objective:**

Cytochrome P450 2C19 (CYP2C19) plays an vital role in the course of cardiovascular and cerebrovascular diseases by affecting lipid metabolism. Triglyceride-glucose (TyG) is a comprehensive index composed of triglyceride and blood glucose, has relationship with some diseases. There was no research report on the association *CYP2C19* polymorphisms, TyG with premature cerebral infarction (CI) (onset ≤ 65 years old) susceptibility.

**Methods:**

This study retrospectively analyzed 1953 CI patients aged ≤ 65 years old from December 2018 to March 2024, and 1919 age-matched individuals with non-CI as controls. The relationship between *CYP2C19* polymorphisms, TyG and premature CI risk were analyzed.

**Results:**

The proportion of hypertension, and diabetes mellitus in patients with premature CI was higher than those in controls. The serum total cholesterol (TC), triglycerides (TG), low-density lipoprotein-cholesterol (LDL-C), and TyG levels in patients with premature CI were significantly higher than those in controls (all *p* < 0.05). The patients had lower *CYP2C19* *1 allele frequency (63.3% vs. 69.6%, *p* < 0.001) and higher *CYP2C19* *2 allele frequency (31.3% vs. 25.4%, *p* < 0.001) than controls. Logistic regression analysis showed that smoking history (odds ratio (OR): 1.193, 95% confidence interval (CI): 1.002–1.422, *p* = 0.048), hypertension (OR: 3.371, 95% CI: 2.914–3.898, *p* < 0.001), diabetes mellitus (OR: 1.911, 95% CI: 1.632–2.237, *p* < 0.001), *CYP2C19* intermediate metabolizer (IM) + poor metabolizer (PM) phenotypes (OR: 1.424, 95% CI: 1.243–1.631, *p* < 0.001), and dyslipidemia (OR: 1.294, 95% CI: 1.077–1.554, *p* = 0.006) were independent risk factors for premature CI.

**Conclusions:**

History of smoking, hypertension, diabetes mellitus, dyslipidemia, and *CYP2C19* IM + PM phenotypes were independently associated with premature CI susceptibility.

## Introduction

Cerebrovascular disease is a kind of disease caused by cerebral vascular disease [1, 2]. Cerebral infarction (CI), also known as ischemic stroke, is a cerebrovascular disease caused by cerebral atherosclerosis and blood circulation disorder in brain tissue, which leads to neurological dysfunction [3]. Due to its high incidence and permanent disability, CI has been recognized as a worldwide public health problem, resulting in a significant economic burden worldwide [4]. Globally, more than 2 million young people suffer from CI each year [5]. The elderly are the main patients of CI; however, it has been found that the age of onset of CI is gradually getting younger in recent years [6, 7]. Studies have reported that young CI patients account for 10–14% of all CI patients [7–9]. Premature (early onset) CI brings heavy burden to society and family, and it is of great significance to study the risk factors of premature CI.

Atherosclerosis (AS) is the main cause of cardiovascular and cerebrovascular diseases. AS is a sustained chronic vascular inflammatory response caused by multiple factors, mainly involving dyslipemia [10]. The disorder of lipid metabolism and the increase of serum lipid levels are the main factors causing AS [11, 12]. When lipids increase, blood viscosity will increase and blood flow rate will slow down, and platelets are easy to adhere to the intima of diseased blood vessels [13]. In addition, hemodynamic changes caused by hypertension are also risk factors for the occurrence of AS [14]. Several studies have found that elevated remnant cholesterol is significantly associated with the risk of ischemic stroke [15, 16]. The study on the association between serum lipid index and the occurrence of premature CI is of great significance to further guide clinical practice. Moreover, insulin resistance (IR) is a metabolic disorder caused by the impaired ability of tissue to respond to insulin stimulation, especially manifested by the disorder of glucose and lipid metabolism [17]. And some studies have linked blood glucose levels to stroke risk [18, 19]. Triglyceride-glucose (TyG) index is a comprehensive index composed of triglyceride and fasting blood glucose (FBG) [20]. A number of studies have shown that TyG index has a good potential in predicting the risk of cardiovascular and cerebrovascular diseases [21, 22]. At present, no relationship between TyG index and the risk of early-onset CI has been reported.

In addition, the progression of atherosclerosis may also be affected by cytochrome P450 (CYP450) enzymes [23, 24]. Specifically speaking, the metabolites of arachidonic acid (AA) endodermal hyperpolarized factor (EDHF) catalyzed by CYP450 are the most important causes of vascular endothelial relaxation [25, 26]. Moreover, reactive oxygen species (ROS) are produced in coronary endothelial cells during the reaction catalyzed by CYP450, which can inhibit the vascular relaxation mediated by nitric oxide (NO) [27]. CYP450 2C19 (CYP2C19) is one of the important members of the CYP450 family [28]. *CYP2C19* *2 (single-nucleotide polymorphisms (SNPs) rs4244285, 681G > A) and *3 (SNP rs4986893, 636G > A) are the two most important loss-of-function alleles of CYP2C19 [29, 30]. *CYP2C19* can be divided into six genotypes: *1/*1, *1/*2, *1/*3, *2/*2, *2/*3, and *3/*3 [31]. According to the genotypes of *CYP2C19*, the enzyme activity types of CYP2C19 can be divided into three phenotypes: extensive metabolizer (EM) (CYP2C19*1/*1), intermediate metabolizer (IM) (CYP2C19*1/*2, and *1/*3), and poor metabolizer (PM) (CYP2C19*2/*2, *2/*3, and *3/*3)) [32, 33].

As a controllable and preventable disease, early identification and intervention of risk factors for cerebral infarction are particularly important for disease prevention and control, especially for young and middle-aged people. The purpose of this study was to investigate the association *CYP2C19* polymorphisms, serum lipid levels with premature CI susceptibility.

## Materials and methods

### Participants

Premature CI was defined as a cerebral infarction that occurred with age ≤ 65 years old. This study retrospectively analyzed 1953 CI patients aged ≤ 65 years old admitted to Meizhou People’s Hospital from December 2018 to March 2024. And 1919 age-matched individuals with non-CI who underwent physical examination in Meizhou People’s Hospital during the same period were used as controls. The diagnosis of CI was in accordance with the criteria set by the Chinese Guidelines for Ischemic Stroke [34], and all were confirmed by computed tomography (CT) or magnetic resonance imaging (MRI) examination.

**Fig. 1:**
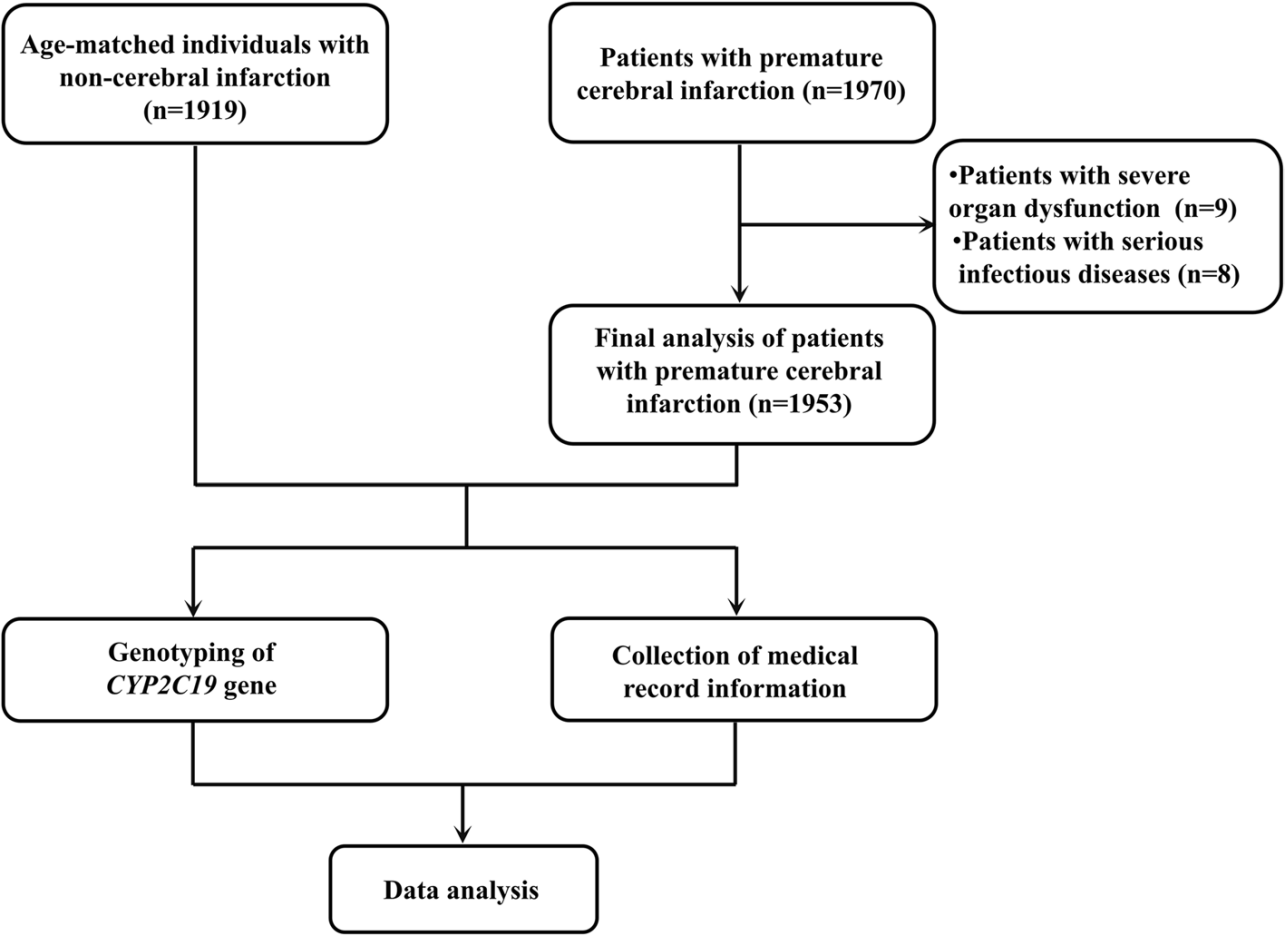
The flowchart of this study

Inclusion criteria of patients: (1) patients diagnosed with CI; (2) patients who performed *CYP2C19* gene polymorphisms; (3) age ≥ 18 years and ≤ 65 years old; and (4) the clinical information of the patients were complete. Exclusion criteria of patients: (1) hemorrhagic CI, and asymptomatic CI; (2) with severe organ dysfunction; and (3) with serious diseases, such as malignant tumors, and severe infections. The inclusion criteria of the controls as follows: (1) non-CI participants who had been performed *CYP2C19* gene polymorphisms test; (2) individuals with ≥ 18 years and ≤ 65 years old; and (3) with complete information. Exclusion criteria of the controls: (1) congenital heart disease, cardiomyopathy, or congestive heart failure; (2) with severe organ dysfunction and serious infectious diseases; and (3) with malignant tumor. This study was supported by the Ethics Committee of the Meizhou People’s Hospital. The flowchart of this study is shown in Fig. 1.

### Data collection and *CYP2C19 *genotyping

Clinical data collected including gender, body mass index (BMI), history of smoking, history of alcohol consumption, hypertension, diabetes mellitus, laboratory and imaging data. In this study, non-elderly people (young and middle-aged) were defined as ≤ 65 years old based on the World Health Organization (WHO) criteria [35]. According to Chinese standards, BMI was divided into three grades: <18.5 kg/m^2^, 18.5–23.9 kg/m^2^, and ≥ 24.0 kg/m^2^ [36, 37]. A diagnosis of dyslipidemia can be made if one of the following conditions is met: (1) total cholesterol (TC) ≥ 6.22 mmol/L), (2) triglyceride (TG) ≥ 2.26 mmol/L, or (3) low-density lipoprotein-cholesterol (LDL-C) ≥ 4.14 mmol/L, according to the Guidelines for the Prevention and Control of Dyslipidemia in Chinese Adults [38, 39]. The TyG index was calculated as Ln[TG (mmol/L)×FBG (mmol/L)/2] [40, 41]. Genomic DNA was extracted from venous blood collected from EDTA anticoagulant collection vessels using a blood DNA isolation kit (Qiagen GmbH, Germany). *CYP2C19* genotyping was performed as previously described [31, 42].

### Statistical analysis

All statistical analysis were performed using SPSS statistical software version 26.0 (IBM Inc., USA). Continuous variables were compared using either Student’s t-test, the Mann-Whitney U test or analysis of variance (ANOVA). Genotype composition ratios and allele frequencies between groups were analyzed using *Chi*-square test or Fisher’s exact test. Hardy-Weinberg equilibrium in the patients and controls was evaluated by *Chi*-square test. Receiver operating characteristic (ROC) curve analysis was used to determine the optimal cutoff value of TyG index to distinguish patients and controls. Logistic regression analysis was applied to examine the relationship of *CYP2C19* phenotypes, TyG index and premature CI risk. *p* < 0.05 was considered to represent statistical significance.

## Results

### Characteristics of subjects

A total of 3872 subjects were included in this study, of which 2521(65.1%) were men and 1351(34.9%) were women. Among them, 1067(27.6%), 233(6.0%), 2477(64.0%), 1352(34.9%) had a history of smoking, drinking, hypertension, and diabetes mellitus, respectively, and 2078(53.7%) were overweight. There was significant difference in the distribution of BMI (*p* = 0.001) between patients with premature CI and controls. The proportion of hypertension (77.7% vs. 50.0%, *p* < 0.001), and diabetes mellitus (43.4% vs. 26.3%, *p* < 0.001) in patients with premature CI was higher than those in controls, respectively. The serum TC, TG, LDL-C, and TyG levels in patients with premature CI were significantly higher than those in controls (all *p* < 0.05) (Table 1).

**Table 1:** Comparison of clinical features among premature cerebral infarction patients and controls

Variables	Total (*n* = 3872)	Controls (*n* = 1919)	Patients (*n* = 1953)	*p* values
Gender				
Male, n(%)	2521(65.1%)	1224(63.8%)	1297(66.4%)	0.092
Female, n(%)	1351(34.9%)	695(36.2%)	656(33.6%)	
BMI (kg/m^2^)				
< 18.5, n(%)	114(2.9%)	75(3.9%)	39(2.0%)	0.001
18.5–23.9, n(%)	1680(43.4%)	837(43.6%)	843(43.2%)	
≥ 24.0, n(%)	2078(53.7%)	1007(52.5%)	1071(54.8%)	
History of smoking				
No, n(%)	2805(72.4%)	1403(73.1%)	1402(71.8%)	0.369
Yes, n(%)	1067(27.6%)	516(26.9%)	551(28.2%)	
History of alcohol consumption				
No, n(%)	3639(94.0%)	1781(92.8%)	1858(95.1%)	0.002
Yes, n(%)	233(6.0%)	138(7.2%)	95(4.9%)	
Hypertension				
No, n(%)	1395(36.0%)	960(50.0%)	435(22.3%)	< 0.001
Yes, n(%)	2477(64.0%)	959(50.0%)	1518(77.7%)	
Diabetes mellitus				
No, n(%)	2520(65.1%)	1415(73.7%)	1105(56.6%)	< 0.001
Yes, n(%)	1352(34.9%)	504(26.3%)	848(43.4%)	
Levels of lipid parameters				
TC, mmol/L, median (P25, P75)	4.60 (3.90, 5.39)	4.53 (3.85, 5.23)	4.68 (3.94, 5.54)	< 0.001
TG, mmol/L, median (P25, P75)	1.52 (1.10, 2.19)	1.45 (1.07, 2.09)	1.59 (1.14, 2.27)	< 0.001
LDL-C, mmol/L, median (P25, P75)	2.61 (2.08, 3.21)	2.44 (1.98, 2.99)	2.81 (2.21, 3.39)	< 0.001
TyG, median (P25, P75)	1.56 (1.13, 2.05)	1.50 (1.09, 1.97)	1.60 (1.16, 2.11)	< 0.001

*BMI* Body mass index, *TC* Total cholesterol, *TG* Triglycerides, *LDL-C* Low-density lipoprotein-cholesterol, *TyG* Triglyceride glucose index, *P25* 25th percentile, *P75* 75th percentile

### Comparison of the *CYP2C19* genotypes and alleles between patients with premature CI and controls

The *CYP2C19* genotype distribution of the subjects included in this study conforms to the Hardy-Weinberg equilibrium (*χ*^2^ = 7.911, *p* = 0.094). The proportion of individuals carried *CYP2C19* *1/*1, *1/*2, *1/*3, *2/*2, *2/*3, and *3/*3 genotype was 43.3%, 39.5%, 6.8%, 7.2%, 3.0%, and 0.3%, respectively. There were 1676 (43.3%), 1794 (46.3%), and 402 (10.4%) individuals with *CYP2C19* EM, IM, and PM phenotype, respectively. The proportions of the *CYP2C19* *1/*2 genotype (41.7% vs. 37.3%, *p* = 0.005) and *CYP2C19* *2/*2 genotype (8.8% vs. 5.5%, *p* < 0.001) were higher, and the frequency of the *CYP2C19* *1/*1 genotype (39.0% vs. 47.6%, *p* < 0.001) was lower in patients with premature CI than those in controls. The *CYP2C19* *1 allele frequency was lower (63.3% vs. 69.6%, *p* < 0.001) and *CYP2C19* *2 allele frequency was higher (31.3% vs. 25.4%, *p* < 0.001) in patients with premature CI than those in controls (Table 2).

**Table 2:** Comparison of the *CYP2C19* genotypes and alleles between patients with premature cerebral infarction and controls

CYP2C19 phenotypes	CYP2C19 genotypes/alleles	Total (*n* = 3872)	Controls (*n* = 1919)	Patients (*n* = 1953)	χ^2^	*p* values
	Genotypes					
Extensive metabolizer	*1/*1	1676(43.3%)	914(47.6%)	762(39.0%)	29.243	< 0.001
Intermediate metabolizer	*1/*2	1530(39.5%)	715(37.3%)	815(41.7%)	8.098	0.005
	*1/*3	264(6.8%)	129(6.7%)	135(6.9%)	0.055	0.848
Poor metabolizer	*2/*2	277(7.2%)	105(5.5%)	172(8.8%)	16.211	< 0.001
	*2/*3	115(3.0%)	50(2.6%)	65(3.3%)	1.754	0.218
	*3/*3	10(0.3%)	6(0.3%)	4(0.2%)	0.437	0.545
	Alleles					
	*1	5146(66.5%)	2672(69.6%)	2474(63.3%)	34.259	< 0.001
	*2	2199(28.4%)	975(25.4%)	1224(31.3%)	33.509	< 0.001
	*3	399(5.2%)	191(5.0%)	208(5.3%)	0.481	0.504
	HWE (χ^2^, *P*)	χ^2^ = 7.911, *p* = 0.094	χ^2^ = 5.569, *p* = 0.234	χ^2^ = 5.175, *p* = 0.270		

*CYP2C19* Cytochrome P450 2C19, *HWE* Hardy Weinberg Equilibrium

### Comparison of clinical characteristics of individuals with different *CYP2C19* phenotypes

The proportion of individuals with diabetes mellitus in *CYP2C19* IM group was higher than those in *CYP2C19* EM and PM groups (41.5% vs. 33.3% and 34.9%, *p* = 0.008). The individuals with *CYP2C19* EM phenotype had higher TC level (4.72 (4.00, 5.54) vs. 4.56 (3.86, 5.32) and 4.38 (3.67, 4.97), *p* < 0.001), and LDL-C level (2.66 (2.15, 3.32) vs. 2.60 (2.06, 3.18) and 2.39 (1.91, 3.01), *p* < 0.001) than those with *CYP2C19* IM phenotype and PM phenotype, respectively (Table 3).

**Table 3:** Comparison of clinical characteristics of individuals with different CYP2C19 phenotypes

Variables	Extensive metabolizer (*n* = 1676)	Intermediate metabolizer (*n* = 1794)	Poor metabolizer (*n* = 402)	*p* values
Gender				
Male, n(%)	1022(61.0%)	1226(68.3%)	273(67.9%)	< 0.001 (χ^2^=22.214)
Female, n(%)	654(39.0%)	568(31.7%)	129(32.1%)	
BMI (kg/m^2^)				
< 18.5, n(%)	53(3.2%)	45(2.5%)	16(4.0%)	0.553 (χ^2^=3.027)
18.5–23.9, n(%)	727(43.4%)	779(43.4%)	174(43.3%)	
≥ 24.0, n(%)	896(53.5%)	970(54.1%)	212(52.7%)	
History of smoking				
No, n(%)	1239(73.9%)	1279(71.3%)	287(71.4%)	0.193 (χ^2^=3.257)
Yes, n(%)	437(26.1%)	515(28.7%)	115(28.6%)	
History of alcohol consumption				
No, n(%)	1575(94.0%)	1684(93.9%)	380(94.5%)	0.904 (χ^2^=0.253)
Yes, n(%)	101(6.0%)	110(6.1%)	22(5.5%)	
Hypertension				
No, n(%)	617(36.8%)	649(36.2%)	129(32.1%)	0.204 (χ^2^=3.172)
Yes, n(%)	1059(63.2%)	1145(63.8%)	273(67.9%)	
Diabetes mellitus				
No, n(%)	1118(66.7%)	1167(65.1%)	235(58.5%)	0.008 (χ^2^=9.709)
Yes, n(%)	558(33.3%)	627(34.9%)	167(41.5%)	
Levels of lipid parameters				
TC, mmol/L, median (P25, P75)	4.72 (4.00, 5.54)	4.56 (3.86, 5.32)	4.38 (3.67, 4.97)	< 0.001
TG, mmol/L, median (P25, P75)	1.53 (1.10, 2.20)	1.52 (1.10, 2.16)	1.50 (1.09, 2.21)	0.862
LDL-C, mmol/L, median (P25, P75)	2.66 (2.15, 3.32)	2.60 (2.06, 3.18)	2.39 (1.91, 3.01)	< 0.001
TyG, median (P25, P75)	1.57 (1.13, 2.05)	1.55 (1.12, 2.05)	1.57 (1.09, 2.10)	0.800

*BMI* Body mass index, *TC* Total cholesterol, *TG* Triglycerides, *LDL-C* Low-density lipoprotein-cholesterol, *TyG* Triglyceride glucose index, *P25* 25th percentile, *P75* 75th percentile

### Logistic regression analysis of risk factors for premature CI

When premature CI was considered as the endpoint of TyG level, the cut-off value of TyG was 1.805 using ROC curve analysis. The results of univariate regression logistic analysis showed that hypertension (yes vs. no, odds ratio (OR): 3.493, 95% confidence interval (CI): 3.039–4.015, *p* < 0.001), diabetes mellitus (yes vs. no, OR: 2.155, 95% CI: 1.882–2.467, *p* < 0.001), *CYP2C19* IM phenotype (IM phenotype vs. EM phenotype, OR: 1.350, 95% CI: 1.181–1.543, *p* < 0.001) and PM phenotype (PM phenotype vs. EM phenotype, OR: 1.795, 95% CI: 1.439–2.241, *p* < 0.001), dyslipidemia (yes vs. no, OR: 1.404, 95% CI: 1.220–1.615, *p* < 0.001), and high TyG level (≥ 1.805 vs. <1.805, OR: 1.451, 95% CI: 1.271–1.656, *p* < 0.001) were significantly associated with premature CI. Multivariate regression logistic analysis showed that history of smoking (yes vs. no, OR: 1.193, 95% CI: 1.002–1.422, *p* = 0.048), hypertension (yes vs. no, OR: 3.371, 95% CI: 2.914–3.898, *p* < 0.001), diabetes mellitus (yes vs. no, OR: 1.911, 95% CI: 1.632–2.237, *p* < 0.001), *CYP2C19* IM + PM phenotype (IM + PM phenotype vs. EM phenotype, OR: 1.424, 95% CI: 1.243–1.631, *p* < 0.001), and dyslipidemia (yes vs. no, OR: 1.294, 95% CI: 1.077–1.554, *p* = 0.006) were independent risk factors for premature CI (Table 4).

**Table 4:** Logistic regression analysis of risk factors for premature cerebral infarction

Variables	Univariate OR (95% CI)	*p* values	Multivariate OR (95% CI)	*p* values
Gender (Male vs. Female)	0.891 (0.780–1.017)	0.086	0.931 (0.795–1.091)	0.377
BMI (kg/m^2^)				
18.5–23.9	1.000 (reference)	-	1.000 (reference)	-
< 18.5	0.516 (0.347–0.769)	0.001	0.637 (0.418–0.971)	0.036
≥ 24.0	1.056 (0.929–1.201)	0.407	0.819 (0.712–0.942)	0.005
History of smoking (Yes vs. No)	1.069 (0.928–1.230)	0.357	1.193 (1.002–1.422)	0.048
History of alcoholism (Yes vs. No)	0.660 (0.504–0.864)	0.002	0.585 (0.433–0.791)	< 0.001
Hypertension (Yes vs. No)	3.493 (3.039–4.015)	< 0.001	3.371 (2.914–3.898)	< 0.001
Diabetes mellitus (Yes vs. No)	2.155 (1.882–2.467)	< 0.001	1.911 (1.632–2.237)	< 0.001
*CYP2C19* phenotypes				
Extensive metabolizer	1.000 (reference)	-	1.000 (reference)	-
Intermediate metabolizer	1.350 (1.181–1.543)	< 0.001	1.367 (1.186–1.575)	< 0.001
Poor metabolizer	1.795 (1.439–2.241)	< 0.001	1.716 (1.355–2.172)	< 0.001
Intermediate metabolizer + Poor metabolizer	1.421 (1.251–1.615)	< 0.001	1.424 (1.243–1.631)	< 0.001
Dyslipidemia (Yes vs. No)	1.404 (1.220–1.615)	< 0.001	1.294 (1.077–1.554)	0.006
TyG (≥ 1.805 vs. <1.805)	1.451 (1.271–1.656)	< 0.001	0.899 (0.744–1.085)	0.266

*BMI* Body mass index, *TC* Total cholesterol, *TG* Triglycerides, *LDL-C* Low-density lipoprotein-cholesterol, *TyG* Triglyceride glucose index

## Discussion

The main causes of CI are poor blood flow in different brain areas caused by various reasons, resulting in necrotic lesions of hypoxic-ischemic brain tissue, which further leads to neurological impairment such as coma and other clinical symptoms. This study analyzed the risk factors of premature CI and found that *CYP2C19* IM + PM phenotype, history of smoking, hypertension, diabetes mellitus, and dyslipidemia were risk factors for premature CI. As one of the important drug metabolizing enzymes in human body, CYP450 is one of the major catalytic enzymes involved in lipid metabolism [43]. The products of CYP450 mainly include epoxy fatty acids and AA, which are important lipid signaling molecules involved in biological regulation [44, 45]. CYP2C19 plays an important role in the metabolism of AA and influences the development of cerebral atherosclerosis by regulating lipid metabolism, blood pressure, and even vascular inflammation.

The relationship between *CYP2C19* gene polymorphisms and ischemic encephalopathy has been reported. Shi et al. found that CYP2C19 IM phenotype was dominant in CI patients, and patients with the CYP2C19 IM phenotype were more likely to develop CI [46]. Fan et al. found that *CYP2C19* gene polymorphism is associated with an increased risk of CI in elderly [47]. Carriers of *CYP2C19* loss-of-function variants have a 1.6 times greater risk of ischemic stroke than non-carriers [48]. CYP2C19 loss-of-function was associated with increased risk of ischemic stroke after transient ischemic attack [49]. In addition, CYP2C19*2 polymorphism was found in the risk of coronary artery disease (CAD) [50]. In this study, *CYP2C19* IM + PM phenotype was independent risk factor for premature CI.

Traditional risk factors for CI susceptibility is an indisputable fact. Poor glycemic control in diabetic patients may be related to the occurrence of CI [51]. Smoking is a risk factor for ischemic stroke in young adults [52]. Hyperlipidemia, diabetes mellitus, and smoking are risk factors for CI [53]. CI is caused by a variety of factors, in addition to life rules, diet, exercise, mood and other factors, hypertension, diabetes mellitus, and hyperlipidemia also show a trend of younger people, and it may be related to the low treatment rate of hypertension, hyperglycemia and hyperlipidemia in young and middle-aged people.

Brain is one of the organs with the highest fat density among all organs, and various lipid mediators produced after ischemic brain injury can change the inflammatory process and promote dissociation process after ischemic brain injury [54]. It is an indisputable fact that hyperlipidemia is a high risk factor for poor prognosis of CI. Lipids and their metabolites can participate in biological transformation through various pathways such as cyclooxygenase metabolic pathway, lipoxygenase metabolic pathway, phospholipid and lysophospholipid metabolic pathway, and cytochrome P450 pathway [55]. Serum lipids can accumulate continuously in the blood vessel wall and become a regulator of atherosclerosis thrombosis, leading to one of the main reasons for CI [56]. A meta-analysis by Amarenco et al. reported that for every 1mmol/L reduction in LDL-C, the relative risk of stroke was reduced by 21.1% [57]. Lee et al. found that a high TyG index was associated with a poorer prognosis in ischemic stroke patients receiving reperfusion therapy [58]. The results of this study showed no association between TyG and the risk of early-onset CI. The role of TyG index in assessing the risk of cardiovascular and cerebrovascular diseases still needs more research to reveal.

In addition, the proportion of *CYP2C19* EM, IM, and PM phenotype was 43.3%, 46.3%, and 10.4%, respectively, in this study. The results of this study are similar to those of some previous studies [59–61]. Gene polymorphism can show obvious racial differences, and there may also be some differences in *CYP2C19* gene polymorphism, which has certain statistical significance. According to the research results of Toshikazu Jinnai et al., the proportion of EM, IM and PM in Japan is about 44%, 32% and 24% [62]. Pongjarat Nun-Anan et al.. calculated *CYP2C19* genotypes in Thai hepatitis patients, and the metabolites of EM, IM, and PM were 42%, 50%, and 8%, respectively [63]. A study has found that CYP2C19*2 and CYP2C19*3 allele variants significantly increase the probability of ischemic stroke recurrence [64]. Another study has also shown that CYP2C19*2 and *3 variant alleles significantly increase the risk of stroke in CI patients taking clopidogrel antiplatelet during cerebrovascular angiography (DSA) and stent implantation [65].

To the best of our knowledge, this study is the first to report on *CYP2C19* gene polymorphisms and premature CI susceptibility. We believe that *CYP2C19* gene polymorphism can provide valuable auxiliary information for the identification and prevention of premature CI. This study is a retrospective study based on a single medical institution, with a small sample size and limited indicators included in the analysis, and the analytical results of this study may be biased. In addition, this study did not analyze the risk factors of different CI subtypes [66], different vascular sites of CI [67] and their relationship with disease severity. Moreover, the clinical features and prognosis of lacunar CI are different from those of non-lacunar CI [68, 69]. The main causes of the association between the two types are the nature of the arterial disease and the diameter of the artery. The difference of risk factors for lacunar CI and non-lacunar CI deserves to be studied, but it was not studied in this study, which is also one of the shortcomings of this study. Therefore, the predictive value of *CYP2C19* polymorphism in early-onset CI needs to be further verified by multi-center prospective studies.

As a controllable and preventable disease, early identification and early intervention of risk factors for CI are particularly important for disease prevention and control. The premature CI is affected by many factors such as genetic markers, biochemical indexes, and living habits. In the future research, there are some aspects worth paying attention to. First, the same individual has different risk factors in the process of producing pathogenic effects, and what is the interaction pattern and mechanism of these risk factors. Second, the number of risk factors of premature CI are relatively large, and controlling which risk factors can significantly reduce the incidence of CI.

## Conclusion

In summary, history of smoking, hypertension, diabetes mellitus, dyslipidemia, and CYP2C19 IM + PM phenotypes were independently associated with premature CI susceptibility. It means that young and middle-aged adults with *CYP2C19* loss-of-function variants and traditional risk factors (smoking, hypertension, diabetes mellitus, and dyslipidemia) need to be aware of the risk of developing CI.

## Data Availability

No datasets were generated or analysed during the current study.
